# TNFSF15 suppresses VEGF production in endothelial cells by stimulating miR-29b expression *via* activation of JNK-GATA3 signals

**DOI:** 10.18632/oncotarget.11683

**Published:** 2016-08-29

**Authors:** Kun Zhang, Hong-Xing Cai, Shan Gao, Gui-Li Yang, Hui-Ting Deng, Guo-Ce Xu, Jihong Han, Qiang-Zhe Zhang, Lu-Yuan Li

**Affiliations:** ^1^ State Key Laboratory of Medicinal Chemical Biology and College of Pharmacy, Tianjin Key Laboratory of Molecular Drug Research, Nankai University, Tianjin, China; ^2^ Collaborative Innovation Center for Biotherapy, Nankai University, West China Hospital, Sichuan University, Chengdu, China; ^3^ College of Life Sciences, Nankai University, Tianjin, China

**Keywords:** tumor necrosis factor superfamily 15, vascular endothelial cell growth factor, microRNA, GATA3, angiogenesis

## Abstract

Vascular endothelial cell growth factor (VEGF) plays a pivotal role in promoting neovascularization. VEGF gene expression in vascular endothelial cells in normal tissues is maintained at low levels but becomes highly up-regulated in a variety of disease settings including cancers. Tumor necrosis factor superfamily 15 (TNFSF15; VEGI; TL1A) is an anti-angiogenic cytokine prominently produced by endothelial cells in a normal vasculature. We report here that VEGF production in mouse endothelial cell line bEnd.3 can be inhibited by TNFSF15 *via* microRNA-29b (miR-29b) that targets the 3'-UTR of VEGF transcript. Blocking TNFSF15 activity by using either siRNA against the TNFSF15 receptor known as death domain-containing receptor-3 (DR3; TNFRSF25), or a neutralizing antibody 4-3H against TNFSF15, led to inhibition of miR-29b expression and reinvigoration of VEGF production. In addition, we found that TNFSF15 activated the JNK signaling pathway as well as the transcription factor GATA3, resulting in enhanced miR-29b production. Treatment of the cells either with SP600125, an inhibitor of JNK, or with JNK siRNA, led to eradication of TNFSF15-induced GATA3 expression. Moreover, GATA3 siRNA suppressed TNFSF15-induced miR-29b expression. These findings suggest that VEGF gene expression can be suppressed by TNFSF15-stimulated activation of the JNK-GATA3 signaling pathway which gives rise to up-regulation of miR-29b.

## INTRODUCTION

Vascular endothelial cell growth factor (VEGF) is a multifaceted cytokine [1]. It promotes embryonic and postnatal neovascularization [2–5], maintains heterogeneity of endothelial cell and organ [6], repairs ischemic tissues and injured organs [7, 8]. Highly elevated levels of VEGF in pathological tissues, as compared with those in normal tissues, are a major threat in a number of diseases including cancer, rheumatoid arthritis, atherosclerosis, diabetic retinopathy, and sepsis [9–13]. Normally seen at low levels [14–16], VEGF gene is among the first set of genes that respond to tissue hypoxia, triggered by hypoxia-inducible factor-1 [17, 18]. How VEGF gene expression is down-regulated, which has important implications in clinical settings, remains unclear, however.

Tumor necrosis factor superfamily 15 (TNFSF15; also known as VEGI or TL1A), a cytokine largely produced by endothelial cells, is a specific inhibitor of endothelial cell proliferation and angiogenesis [19–21]. TNFSF15 can induce proliferating endothelial cells to undergo apoptosis [22] and thus inhibit angiogenesis [23]. It can also inhibit the differentiation of endothelial progenitor cell into endothelial cell [24] by facilitating the production of a soluble form of VEGF receptor-1 (VEGFR1), which competes with the full length, signal-transmitting form of VEGFR1 for VEGF, thus blocking VEGF activity [25]. The expression of the TNFSF15 gene in endothelial cells is often found at high levels in mature vasculature in normal tissues, it diminishes in angiogenic vasculatures such as in cancers [26–28] and wound tissues [29]. A number of cytokines, noticeably VEGF, can effectively suppress TNFSF15 production [26]. These experimental data indicate that VEGF and TNFSF15 may act as a pair of counter-balancing factors in the maintenance of vascular integrity and regulation of neovascularization.

We report here that TNFSF15 is able to stimulate in endothelial cell the production of a microRNA, miR-29b, whose targets include VEGF mRNA. We also show that TNFSF15 activates the JNK signaling pathway to promote the activation of transcription factor GATA3, which drives the expression of miR-29b, thus the inhibition of VEGF production.

## RESULTS

### TNFSF15 inhibits VEGF expression in mouse endothelial cells

**Figure 1:**
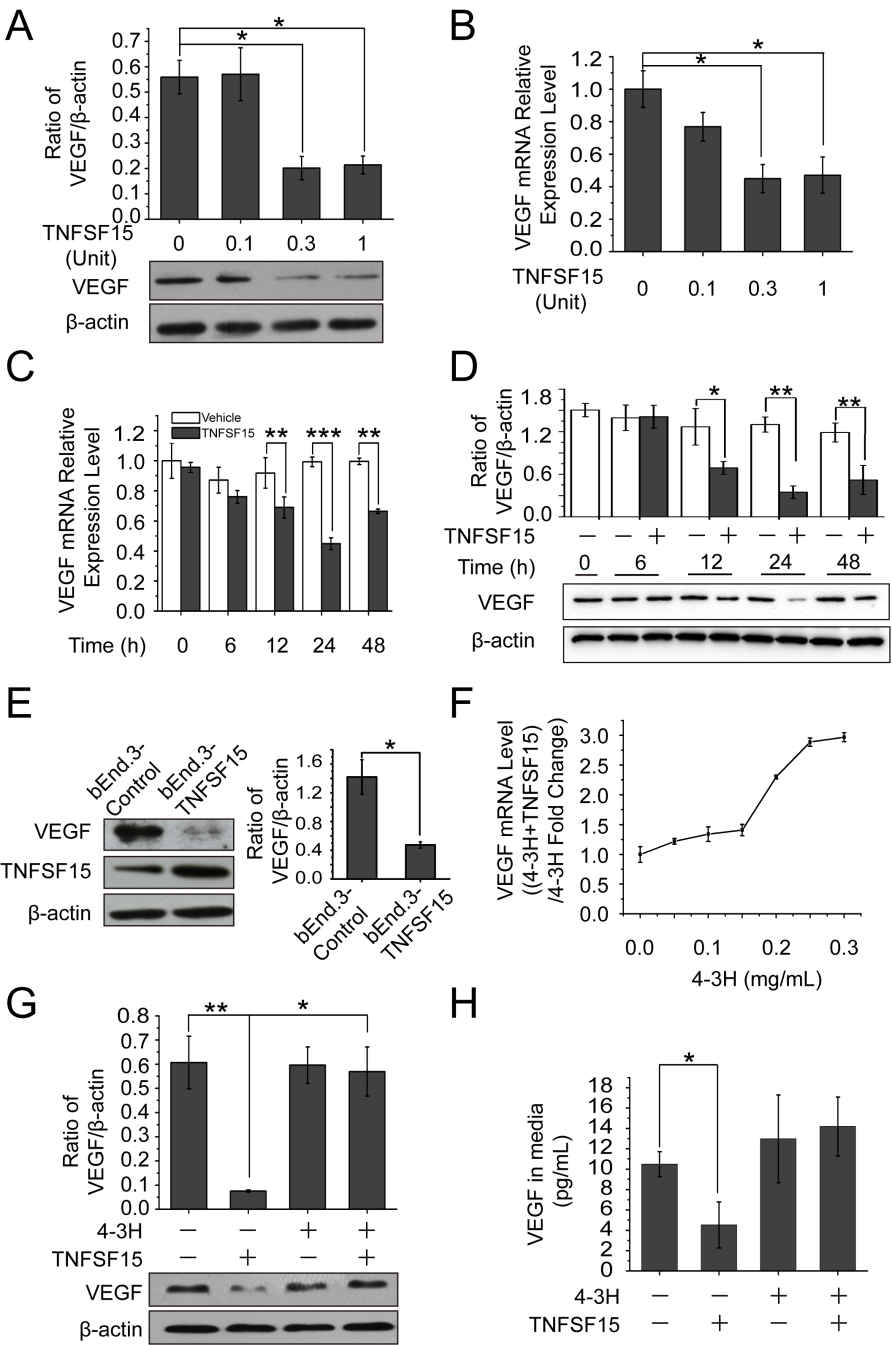
TNFSF15 down-regulates VEGF expression in bEnd.3 cells **A.** Changes in VEGF protein levels in bEnd.3 cells treated with recombinant TNFSF15 treatment at concentrations indicated. Data are mean±SD. *p < 0.05; one-way ANOVA. **B.** Changes in VEGF mRNA levels in bEnd.3 cells following TNFSF15 treatment at indicated concentrations. Data are mean±SD. *p < 0.05; one-way ANOVA. **C.** VEGF mRNA levels in vehicle- (white) or TNFSF15-treated (black) bEnd.3 determined at indicated time intervals by RT-PCR. Data are mean±SD. **p < 0.01; ***p < 0.001; Student's *t*-test. **D.** VEGF protein levels in vehicle- (white) or TNFSF15-treated (black) bEnd.3 at indicated time intervals. Data are mean±SD. *p < 0.05; **p < 0.01; Student's *t*-test. **E.** Western blot analysis of VEGF protein levels in bEnd.3 cells transiently transfected with either the TNFSF15 gene or an empty vector. Values are normalized to β-actin. Data are mean±SD. *p < 0.05; Student's *t*-test. **F.** Changes in VEGF mRNA levels in TNFSF15-treated bEnd.3 in the presence or absence of TNFSF15 neutralizing antibody 4-3H at indicated concentrations. **G.** Changes in VEGF protein levels in vehicle- or TNFSF15-treated bEnd.3 in the presence or absence of 4-3H (0.2 mg/mL). Data are mean±SD. *p < 0.05; **p < 0.01; one-way ANOVA. **H.** Concentrations of secreted VEGF in culture media determined by ELISA following TNFSF15 treatment in the presence or absence of 4-3H. Data are mean±SD. *p < 0.05; Student's *t*-test. Each experiment was performed three times.

We treated the mouse endothelial cell line bEnd.3 with recombinant human TNFSF15, and found that the treatment resulted in a substantial decrease of VEGF production within 24 hrs at both protein and mRNA levels in a TNFSF15 dose-dependent manner (Figures 1A, 1B). VEGF mRNA levels declined within 12 hrs of the treatment, and became about 2-times lower than that in vehicle-treated cells within 24 hrs (Figure 1C) while VEGF protein levels decreased by 3-fold (Figure 1D). Additionally, we transfected these cells with the mouse TNFSF15 gene, and found that the engineered overexpression of TNFSF15 was accompanied by a markedly lowered VEGF expression (Figure 1E). Moreover, we found that the presence of a TNFSF15 neutralizing antibody, 4-3H [25], in the culture media prevented TNFSF15 inhibition of VEGF production at mRNA and protein levels (Figure 1F, 1G); the amount of secreted VEGF in the cell culture media also declined under the experimental conditions (Figure 1H). These findings indicate that TNFSF15 is able to specifically and effectively inhibit VEGF expression in endothelial cells.

### TNFSF15 up-regulates VEGF-targeting miR-29b in bEnd.3 cells

**Figure 2:**
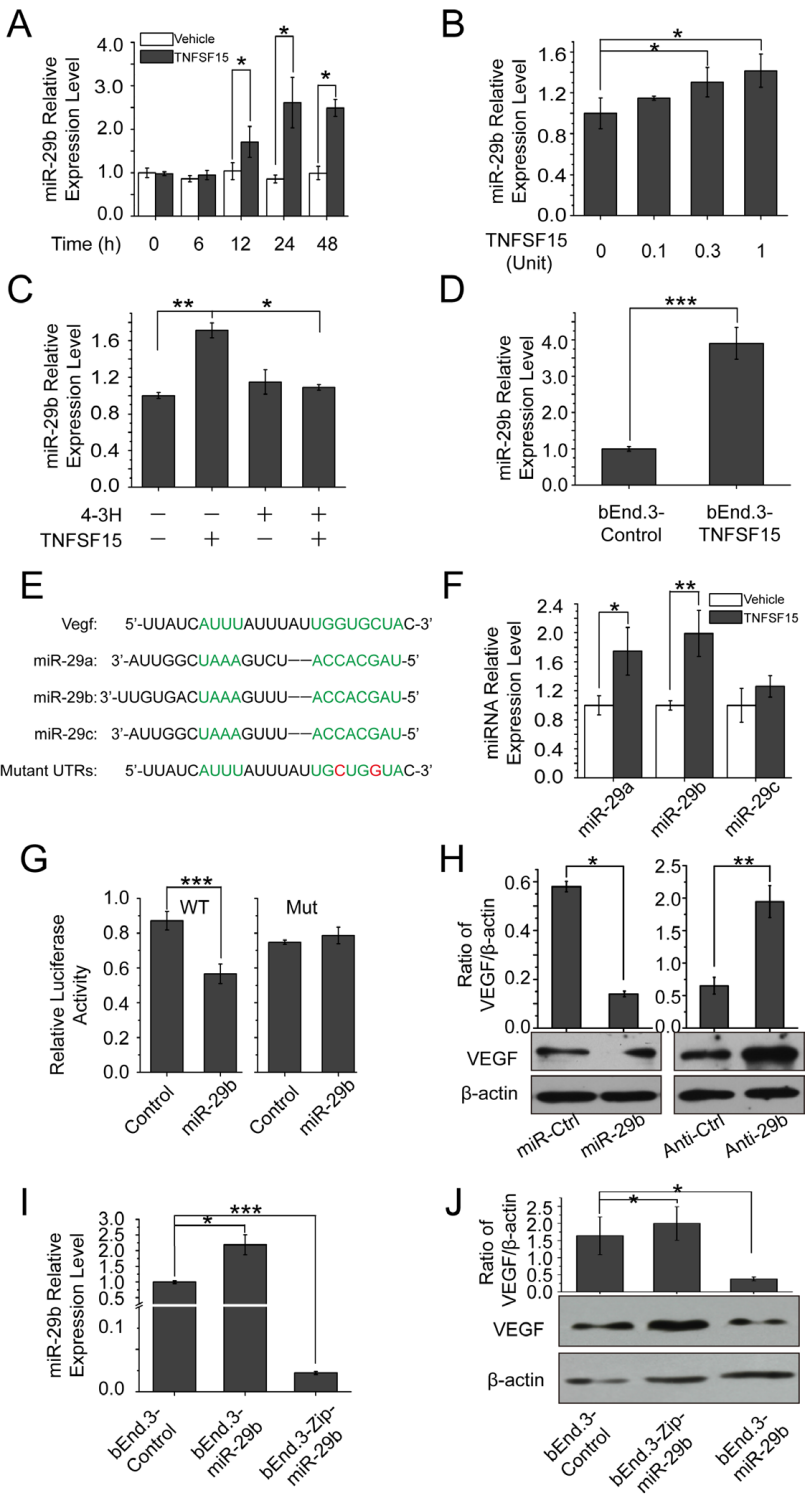
TNFSF15 up-regulates VEGF-targeting miR-29b in bEnd.3 cells **A.** MiR-29b levels in vehicle- (white) or TNFSF15-treated (black) bEnd.3 at indicated time intervals determined by RT-PCR. Data are mean±SD. *p < 0.05; Student's *t*-test. **B.** Changes in miR-29b levels following TNFSF15 treatment at indicated concentrations. Data are mean±SD. *p < 0.05; one-way ANOVA; Student's *t*-test. **C.** Changes in miR-29b levels in TNFSF15-treated bEnd.3 in the presence of 4-3H at indicated concentrations. Data are mean±SD. *p < 0.05; **p < 0.01; one-way ANOVA; **D.** Changes in miR-29b levels in TNFSF15- or vector-transfected bEnd.3 cells. Data are mean±SD. ***p < 0.001; Student's *t*-test. **E.** Sequences of miR-29a, miR-29b, and miR-29c; Seed sequence and the complementary binding sites are in green. The mutations generated within the 3' UTR in red. **F.** Relative expression of miR-29a, miR-29b and miR-29c in vehicle- or TNFSF15-treated bEnd.3 measured by qPCR. Data are mean±SD. *p < 0.05; **p < 0.01; Student's *t*-test. **G.** Regulation of VEGF by miR-29b was confirmed by luciferase reporter and mutagenesis assays. WT indicates wild-type. Mut indicates mutant-type. Data are mean±SD. ***p < 0.001; Student's *t*-test. **H.** Changes of VEGF protein in bEnd.3 transfected with miR-29b and anti-miR-29b mimics. Data are mean±SD. *p < 0.05; **p < 0.01; Student's *t*-test.**I.** Relative levels of miR-29b in bEnd.3 infected with lentivirus encoding miR-ctr, miR-29b or miR-Zip29b, determined by RT-PCR. Data are mean±SD. *p < 0.05; ***p < 0.001; one-way ANOVA. **J.** Changes of VEGF protein levels in bEnd.3 cells infected with lentivirus encoding miR-ctr, miR-29b or miR-Zip29b. Data are mean±SD. *p < 0.05; one-way ANOVA. Each experiment was performed three times.

MicroRNA-29b is known to be involved in the down-regulation of VEGF [30, 31], We found that miR-29b level in bEnd.3 cells increased by 2-times within 24 hrs of TNFSF15 treatment (Figure 2A), and the increase of miR-29b was dose-dependent (Figure 2B). Presence of 4-3H in the culture media prevented TNFSF15 from stimulating miR-29b levels (Figure 2C). Overexpressing mouse TNFSF15 in bEnd.3 cells also led to an up-regulation of miR-29b (Figure 2D). Carrying out computational miRNA target analysis, we identified sequences in the 3'-untranslated region of the VEGF gene (VEGF3′UTR) that are complementary to the sequences of miR-29b, suggesting that VEGF be a molecular target for miR-29b. The miR-29 family consists of three members, miR-29a, -29b and -29c, with the same seed sequence (Figure 2E) [30]. In addition to that of miR-29b, intracellular levels of miR-29a in bEnd.3 also increased in response to TNFSF15 treatment, although to a less extent compared with miR-29b (Figure 2F). To determine whether miR-29b directly targeted to VEGF 3′UTR, we constructed luciferase reporter with WT and point mutations VEGF 3′UTR (WT3′UTR and Mut3′UTR) for miR-29b. We found that co-transfection of miR-29b and WT3′UTR in human embryonic kidney cell line 293T cells resulted in repressed luciferase activity; co-transfection of an unrelated miRNA had little effect (miR-Ctr). Co-transfection of miR-29b and Mut3′UTR, abolished the repression of luciferase activity (Figure 2G). To further demonstrate the impact of miR-29b on VEGF, we transfected miR-29b and anti-miR-29b mimics into bEnd.3 cells. Western blotting showed a significant decrease in VEGF protein level after treatment with the miR-29b mimic, while inhibition of miR-29b by anti-miR-29b increase VEGF protein levels (Figure 2H). We then generated stable miR-29b overexpression cells with lentivirus encoding miR-29b or a scrambled miRNA control and miR-29b knockdown cells using miR-Zip29b lentivirus (Figure 2I). We also examined VEGF expression level in these three cell lines and find that VEGF was down-regulated in bEnd.3-miR-29b cell line and up-regulated in bEnd.3-Zip-miR-29b cell line as compared with bEnd.3-control (Figure 2J). These data indicate that miR-29b directly interact with VEGF 3′UTR, repressing VEGF expression. We therefore reasoned that TNFSF15 regulates VEGF through miR-29b.

### TNFSF15 enhances GATA3 expression to promote miR-29b production

**Figure 3:**
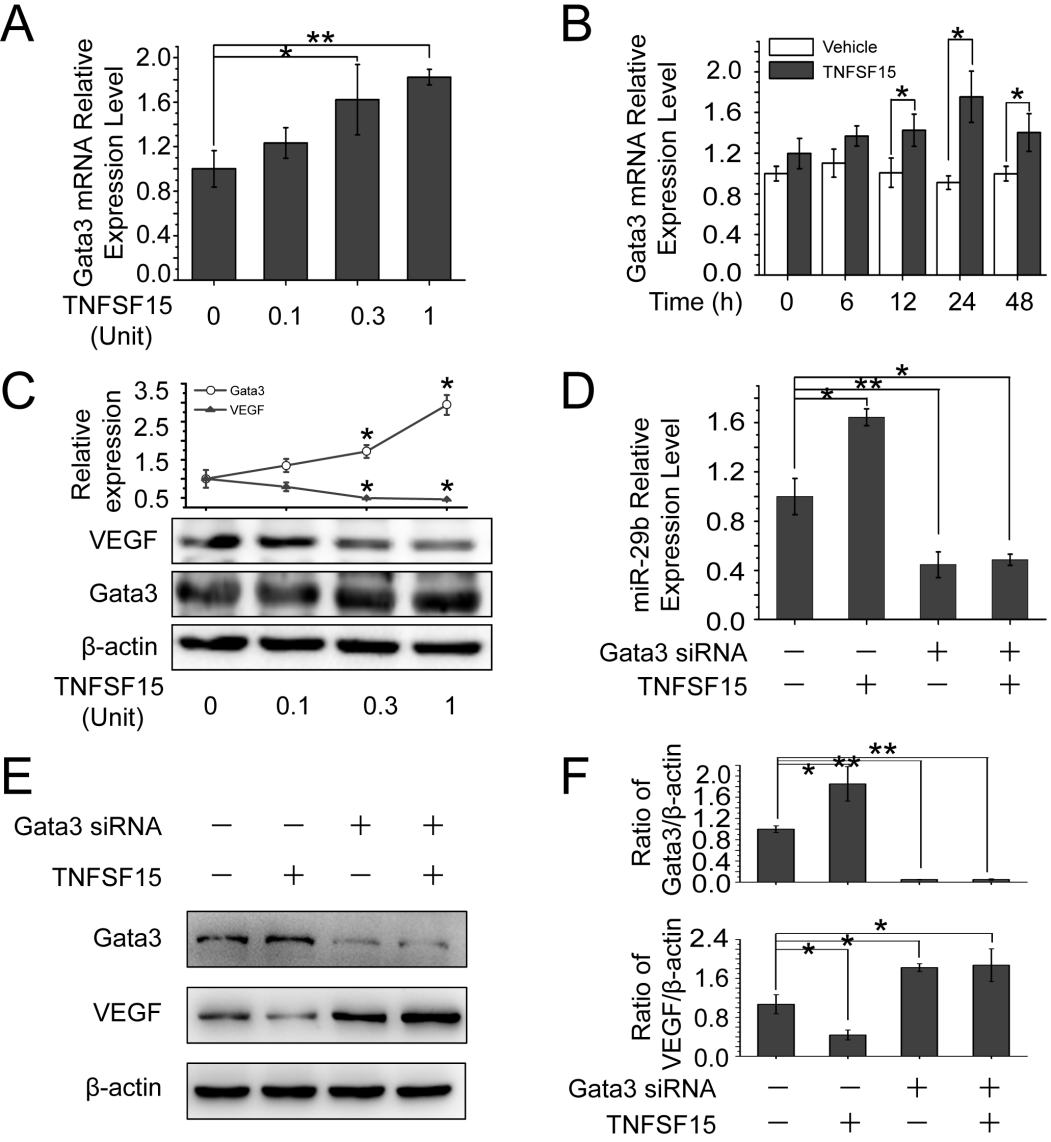
TNFSF15 up-regulates GATA3 expression, which promotes miR-29b production to silencing VEGF **A.** GATA3 mRNA levels in bEnd.3 cells treated with TNFSF15 at indicated concentrations. Data are mean±SD. *p < 0.05; **p < 0.01; one-way ANOVA. **B.** GATA3 mRNA levels in vehicle- (white) or TNFSF15-treated (black) bEnd.3 at indicated time intervals. Data are mean±SD. *p < 0.05; Student's *t*-test. **C.** Changes of GATA3 and VEGF protein levels in bEnd.3 cells following TNFSF15 treatment at indicated concentrations. Values are normalized to β-actin **D.** Changes of miR-29b levels in response to TNFSF15 treatment in the presence or absence of GATA3 siRNA (160 pmol/mL), determined by RT-PCR. Data are mean±SD. *p < 0.05; one-way ANOVA. **E.** Changes of VEGF protein levels following TNFSF15 treatment (0.3 Unit, 24 hrs) in the presence or absence of GATA3 siRNA (160 pmol /mL). **F.** Densitometry analysis of GATA3 and VEGF protein band intensities shown in panel E. Data are mean±SD. *p < 0.05; **p < 0.01; one-way ANOVA. Each experiment was performed three times.

Since it is known that transcription factor GATA3 promotes miR-29b expression in other types of cells [30], we treated bEnd.3 cells with TNFSF15 and found that GATA3 was up-regulated at both protein and mRNA levels by TNFSF15 (Figures 3A-3C), and this was accompanied by a down-regulation of VEGF (Figure 3C). To determine whether GATA3 was required for miR-29b expression, we treated bEnd.3 with GATA3 siRNA (160 pmol/mL) prior to TNFSF15 treatment, and found that GATA3 gene silencing prevented the TNFSF15-stimulation of up-regulation of miR-29b (Figure 3D). GATA3 siRNA treatment also prevented TNFSF15-induced down-modulation of VEGF (Figure 3E, 3F). These findings indicate that GATA3 activation by TNFSF15 is necessary in miR-29b up-regulation.

### DR3 mediates TNFSF15-stimulated activation of GATA3, up-regulation of miR-29b and down-regulation of VEGF

**Figure 4:**
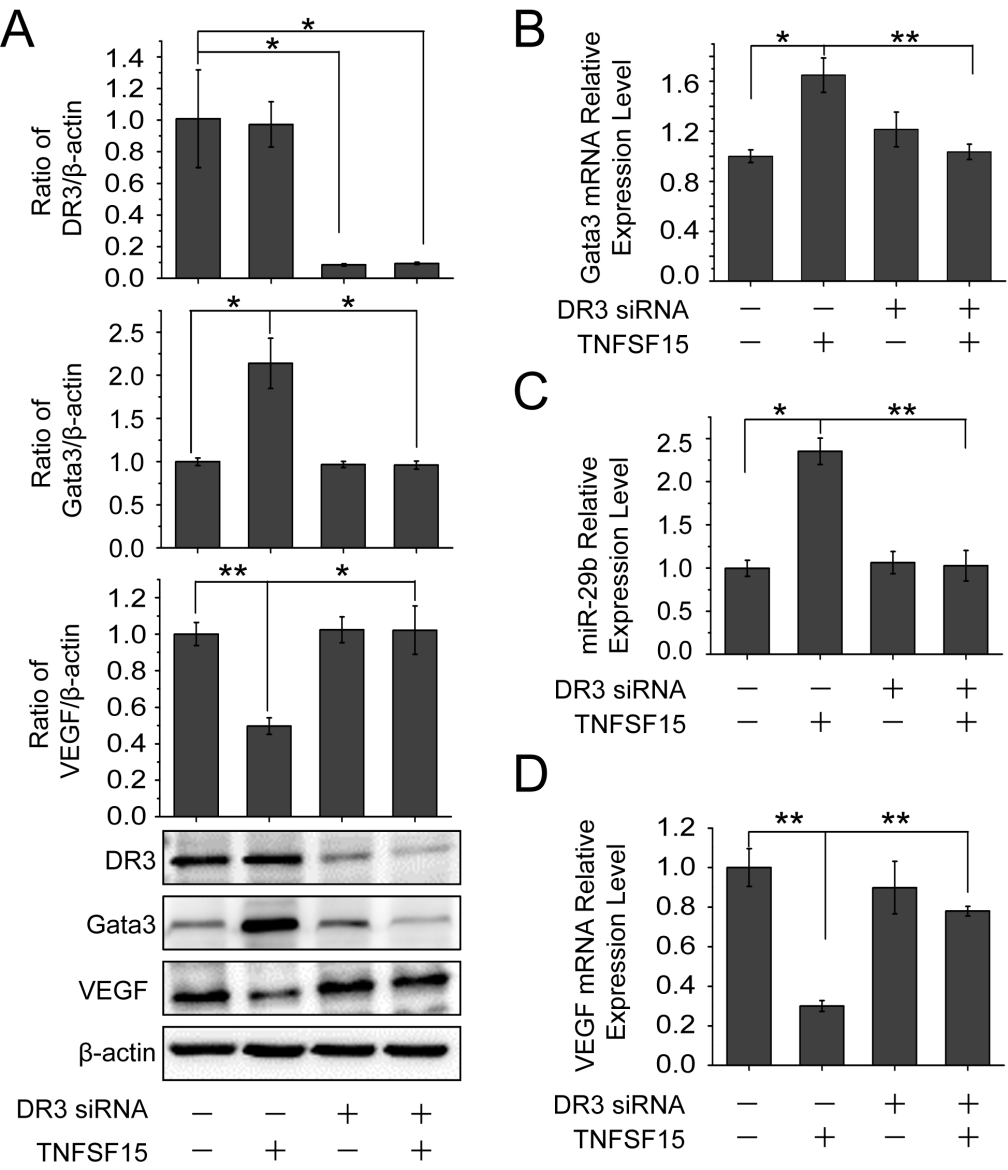
DR3 mediates TNFSF15- stimulate activation of GATA3, up-regulation of miR-29b and down-regulation of VEGF **A.** Changes of GATA3 and VEGF protein levels following TNFSF15 treatment (0.3 Unit, 24 hrs) in the presence or absence of DR3 siRNA (40 pmol/mL), determined by Western blotting analysis. Data are mean±SD. *p < 0.05; **p < 0.01; one-way ANOVA. **B.** Changes of GATA3 mRNA levels in response to TNFSF15 treatment in the presence or absence of DR3 siRNA (40 pmol/mL). Data are mean±SD. *p < 0.05; **p < 0.01; one-way ANOVA. **C.** Changes of miR-29b levels in response to TNFSF15 treatment in the presence or absence of DR3 siRNA (40 pmol/mL). Data are mean±SD. *p< 0.05; **p < 0.01; one-way ANOVA. **D.** Changes of VEGF mRNA levels in response to TNFSF15 treatment in the presence or absence of DR3 siRNA (40 pmol/mL). Data are mean±SD. **p < 0.01; one-way ANOVA; Each experiment was performed three times.

To determine whether the up-regulation of GATA3 and miR-29b was mediated by DR3, the cell surface receptor for TNFSF15, we treated bEnd.3 cells with DR3 siRNA. Western blotting analysis revealed that TNFSF15 was no longer able to stimulate an increase of the GATA3 protein in the cells, or to inhibit VEGF production once the DR3 gene is silenced (Figure 4A). DR3 gene-silencing also resulted in a blockage of TNFSF15-induced up-regulation of GATA3 at mRNA level by using RT-PCR (Figure 4B). Concomitantly, DR3 siRNA treatment resulted in an inhibition of TNFSF15-stimulated miR-29b up-regulation (Figure 4C), as well as the inability of TNFSF15 to inhibit VEGF gene expression at mRNA level (Figure 4D). These findings indicate that DR3 is responsible for mediating TNFSF15 activities that lead to the activation of GATA3, the up-regulation of miR-29b and, consequently, the down-modulation of VEGF gene expression in bEnd.3 cells.

### JNK signaling is involved in TNFSF15-stimulated GATA3 and miR-29b up-regulation

**Figure 5:**
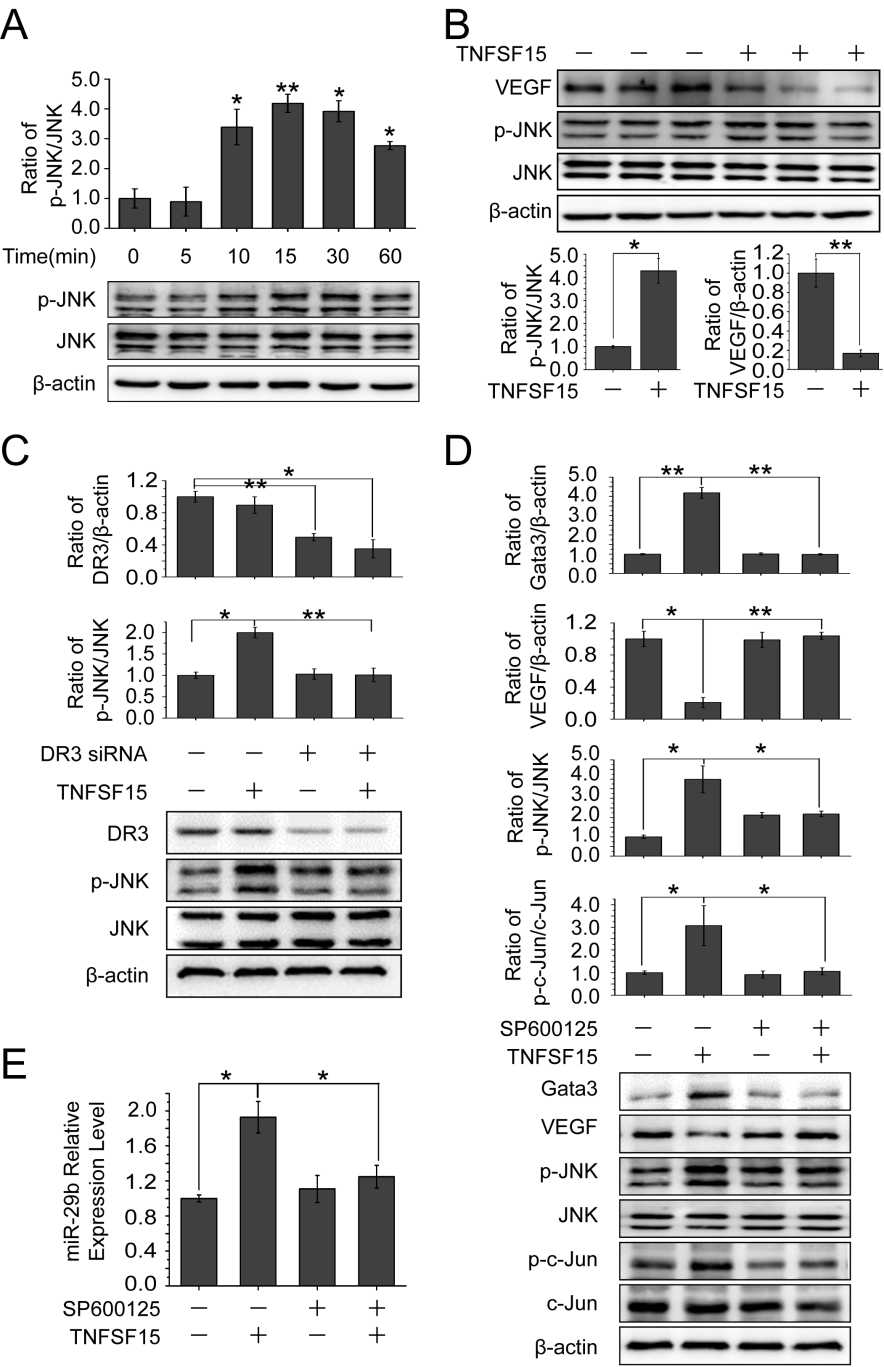
Involvement of JNK signaling pathway in TNFSF15-facilitated GATA3 and miR-29b up-regulation and VEGF down-regulation **A.** Western blotting analysis of phosphorylated JNK proteins following TNFSF15 treatment within 60 min. Data are mean±SD. *p< 0.05; **p < 0.01; one-way ANOVA. **B.** Western blotting analysis of phosphorylated JNK proteins following TNFSF15 treatment after 24 hours. Data are mean±SD. *p < 0.05; **p < 0.01; Student's *t*-test. **C.** Changes of DR3 and phosphorylated JNK protein levels following TNFSF15 treatment (0.3 Unit, 24 hrs) in the presence or absence of DR3 siRNA (40 pmol/mL). Data are mean±SD. *p < 0.05; **p < 0.01; one-way ANOVA. **D.** Effect of SP600125 (50 μM) on TNFSF15-induced VEGF degradation, GATA3 up-regulation, JNK phosphorylation and c-jun phosphorylation. Data are mean±SD. *p < 0.05; **p < 0.01; one-way ANOVA. **E.** Changes of miR-29b levels following TNFSF15 treatment (24 hrs) in the presence or absence of SP600125 (50 μM). Data are mean±SD. *p < 0.05; one-way ANOVA. Each experiment was performed three times.

It is known that TNFSF15 activates JNK pathway in a DR3-dependent manner [32–34]. To find out the role of JNK signaling in TNFSF15-facilitated VEGF down-modulation, we treated bEnd.3 cells with TNFSF15 (0.3 Unit) and evaluated the phosphorylation of JNK. Western blotting analysis of the treated cells indicated that TNFSF15 treatment led to a substantial increase in phosphorylated JNK (p-JNK) within 10 min (Figure 5A). This effect remained profound when measured at 24 hrs post treatment, and coincided with delined VEGF gene expression (Figure 5B). Additionally, silencing the DR3 gene with siRNA (40 pmol/mL) efficiently abolished TNFSF15 activation of JNK (Figure 5C). We then treated bEnd.3 cells with a JNK inhibitor, SP600125 (50 μM, 2 hrs), prior to TNFSF15 treatment, and found that SP600125 effectively inhibited phosphorylation of c-Jun, the transcriptor that transmits JNK signal [35, 36]; SP600125 treatment also completely abolished TNFSF15-induced GATA3 upregulation, and prevented TNFSF15 from inhibiting VEGF production (Figure 5D). Additionally, SP600125 treatment effectively prevented TNFS15-induced miR-29b up-regulation (Figure 5E). These findings support the view that TNFSF15 activates JNK signaling pathway to promote the expression of GATA3, which drives the expression of miR-29b.

As an alternate approach to examine the significance of JNK for TNFSF15-stimulated GATA3 and miR-29b up-regulation, we silenced the JNK gene by using a siRNA molecule that recognizes a common sequence in both JNK1 and JNK2 from both mice and humans [37]. We found that treatment of bEnd.3 cells with the JNK siRNA prevented TNFSF15-induced GATA3 upregulation and resulted in lowered VEGF production (Figure S1A). We also found that JNK siRNA effectively prevented TNFS15-induced miR-29b up-regulation (Figure S1B). These data, which are in good agreement with those obtained with JNK inhibitor SP600125, demonstrated an critical role for JNK in the modulation of TNFSF15-stimulalted expression of GATA3 and miR-29b.

### MiR-29b inhibits angiogenesis *in vitro* and *in vivo*

**Figure 6:**
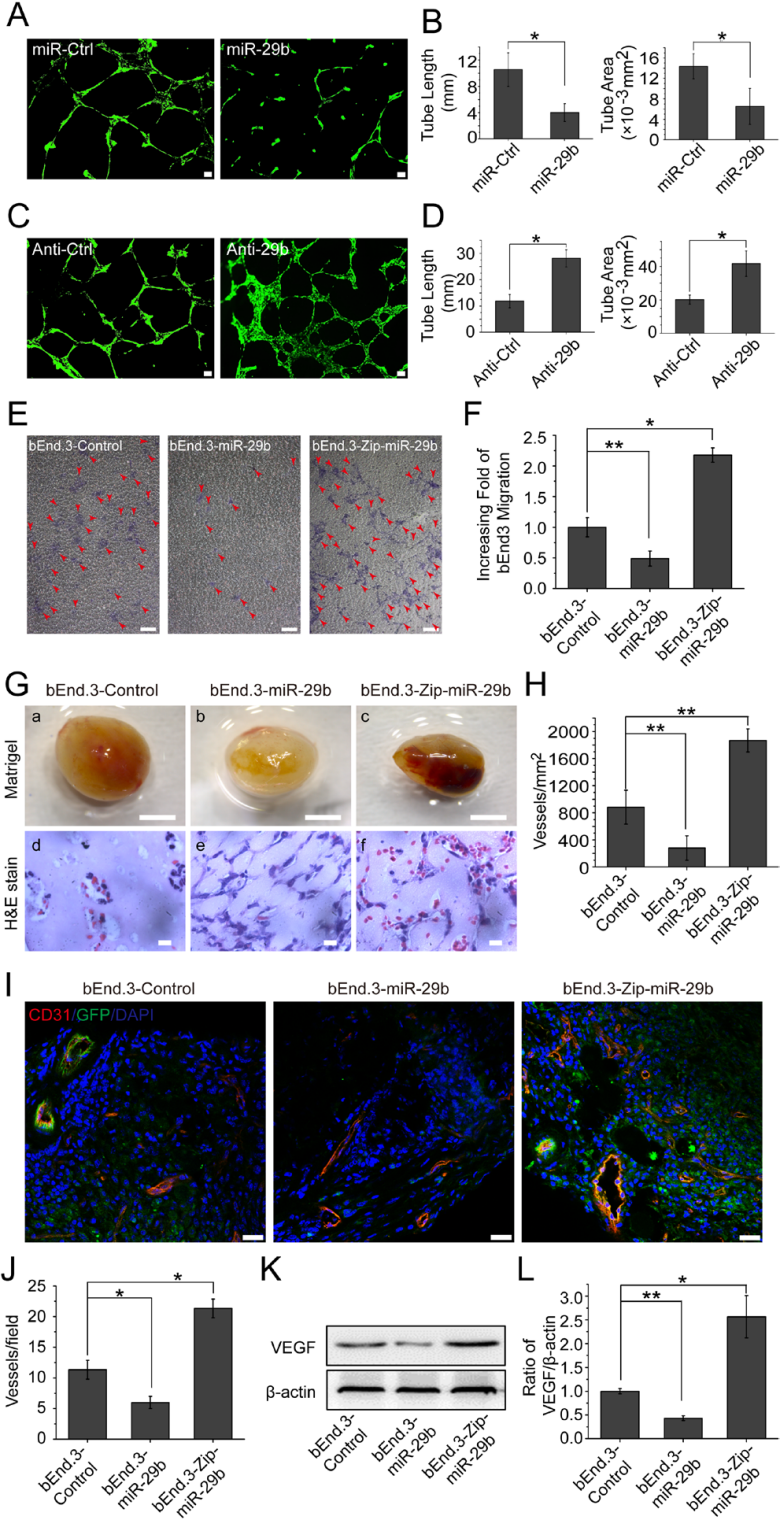
miR-29b inhibits angiogenesis *in vitro* and *in vivo* **A.** Images of capillary-like tubules formed by bEnd.3 cells transfected with miR-Ctrl or miR-29b mimics on Matrigel in 24 hrs after seeding; scale bar, 100 μm. **B.** Average lengths and areas of the capillary-like tubules formed by miR-Ctrl or miR-29b mimics transfected cells. Data are mean±SD of three independent experiments; *p < 0.05; Student's *t*-test. **C.** Images of capillary-like tubules formed by bEnd.3 cells transfected with anti-Ctrl or anti-miR-29b mimics in 24 hrs after seeding; scale bar, 100 μm. **D.** Average lengths and areas of the capillary-like tubules formed by anti-Ctrl or anti-miR-29b mimics-transfected cells. Data are mean±SD of three independent experiments; *p < 0.05; Student's *t*-test. **E.** Images of migrated bEnd.3 cells in transwell assays for miR-control, miR-29b or Zip-miR-29b-transfected cells (arrows); scale bar, 25 μm. **F.** Quantitative analysis of the numbers of migrated cells transfected with miR-control, miR-29b or Zip-miR-29b. Data are mean±SD of three independent experiments; *p < 0.05;**p < 0.01; one-way ANOVA. **G.** Comparison of blood vessel formation in Matrigel plugs on C57BL/6J mice (*n* = 6 per group) of bEnd.3-control (a, d), bEnd.3-miR-29b (b, e), or bEnd.3-Zip-miR-29b (c, f); scale bar for images of Matrigel plugs (a, b, and c): 2 mm; scale bar for H&E-stained cross-sections: 5 μm (d, e and f). **H.** Quantitative analysis of blood vessel densities in H&E-stained sections of miR-control or miR-29b or Zip-miR-29b plugs. Data are mean±SD. **p < 0.01; one-way ANOVA.**I.** Fluorescent confocal microscopic images of frozen sections of Matrigel plugs from miR-control or miR-29b or Zip-miR-29b groups; red, CD31; green, GFP; blue, DAPI; yellow, CD31-GFP double positive; scale bar, 25 μm. **J.** Quantitative analysis of microvessel densities of the gel plugs (*n* = 6 per group) determined by counting the number of CD31-positive vascular structures containing lumens in the fluorescent confocal microscopic images; 3 images from 3 sections for each gel plug were analyzed. Data are mean±SD. *p < 0.05; one-way ANOVA. **K.** Western blotting analysis of VEGF protein levels in the gel plugs. **L.** Densitometric analysis of VEGF protein band intensities shown in panel K; measurements from three independent Western analyses were used. Data are mean±SD. *p < 0.05; **p < 0.01; one-way ANOVA.

To investigate the effect of miR-29b modulation on angiogenesis, we transfected bEnd.3 cells with miR-29b mimics, and found that the miR-29b mimics markedly inhibited bEnd.3 cells to form capillary-like tubules on Matrigel coating (Figure 6A, 6B). On the other hand, when we overexpressed anti-miR-29b in bEnd.3 cells in order to inhibit miR-29b, we found that the cells were more capable of tubule formation (Figure 6C, 6D). In addition, to determine the impact of miR-29b on the ability of bEnd.3 cell to migrate, we infected the cells with Lentivirus-linked miR-29b vector. The results indicated that miR-29b overexpression led to a marked reduction of bEnd.3 cells to migrate through microporous filters (Figure 6E). Furthermore, inhibition of miR-29b by Zip-miR-29b led to more than a doubling of the ability of bEnd.3 cells to migrate (Figure 6F).

To investigate miR-29b modulation of angiogenesis *in vivo*, we injected a Matrigel solution containing bEnd.3-miR-29b cells, bEnd.3-Zip-miR-29b cells, or bEnd.3-control cells subcutaneously on C57BL/6J mice. When retrieved on day 14 post-implantation, we found that the gel plugs contained bEnd.3-Zip-miR-29b cells became highly vascularized compared with those containing bEnd.3-control cells, whereas those contained bEnd.3-miR-29b cell had much fewer blood vessels compared with bEnd.3-control cells (Figure 6G). Analysis of blood vessel densities of the three groups by counting the number of red blood cell-containing blood vessels confirmed the ability of miR-29b to inhibit angiogenesis in this model (Figure 6H). As the cells in this experiment were labeled with green fluorescent protein (GFP), we sectioned the freshly frozen gel plugs, carried out immunostaining for endothelial cell marker CD31, and subjected the sections to confocal microscopic analysis. The new blood vessels were CD31-positive (red) and GPF-positive (green) and therefore appeared yellow (Figure 6I). Quantitative analysis of the microscopic images indicated that, compared with empty vector transfected bEnd.3 cells, miR29b overexpressing cells were much less capable of forming blood vessel, whereas removal of miR-29b by Zip-miR-29b facilitated blood vessel growth (Figure 6J). Moreover, Western blotting analysis of the plugs indicated that VEGF protein levels of the bEnd.3-miR-29b group decreased by 2-fold, compared with the bEnd.3-control group. In contrast, VEGF levels of bEnd.3-Zip-miR-29b group increased by 2.5-fold by the same comparison (Figure 6K, 6L). These findings indicate that miR-29b down-regulation of VEGF expression leads to inhibition of angiogenesis in this animal model.

## DISCUSSION

We show in this study that TNFSF15, a cytokine produced largely by endothelial cells and a potent inhibitor of endothelial cell proliferation, is able to down-regulate VEGF gene expression. This is achieved by TNFSF15-stimulated expression of miR-29b, which marks VEGF mRNA for destruction. Additionally, we demonstrate that DR3, the cell-surface receptor for TNFSF15, mediates the stimulation of miR-29b expression by activating the JNK signaling pathway, which leads to the activation of transcription factor GATA3 that promotes miR-29b expression. The overall outcome of this sequence of signals is an inhibition of VEGF-driven neovascularization.

These findings are interesting because they reveal a new signaling pathway by which the activity of VEGF, possibly the most prominent factor that drives blood vessel growth under a variety of pathological conditions, including inflammation and cancer, can be directly modulated. We showed previously that VEGF effectively down-regulate TNFSF15 expression in ovarian cancer, indicating that TNFSF15 as a negative regulator of blood vessel growth is targeted by VEGF for down-modulation in order to initiate angiogenesis [26]. Taken together, our findings suggest that TNFSF15 and VEGF be a pair of counter-balancing factors that play a pivotal role in the modulation of neovascularization in physiological or pathological settings.

DR3, the cell surface receptor for TNFSF15, has been shown to mediate TNFSF15 activities in T cells, dendritic cells and lymphatic endothelial cells [38–40]. The experimental data from this study indicate that DR3 is also involved in mediating TNFSF15 activity in miR-29b up-regulation and VEGF down-regulation through the activation of JNK signaling pathway, offering a mechanism underlying the promotion of miR-29b by TNFSF15. That JNK inhibitor SP600125 treatment of the cells results in a blockage of TNFSF15-induced GATA3 up-regulation indicates that TNFSF15-activated JNK signaling pathway can be directly linked to the promote the expression of transcription factor GATA3. In agreement with a previous report that GATA3 is able to induce the production of miR-29b by enhancing the activity of the miR-29b promoter [30], we demonstrate in this study that silencing the GATA3 gene results in an abrogation of TNSF15-induced miR-29b up-modulation and subsequent VEGF down-modulation.

In summary, the results of this study bring forward insights into a mechanism through which the down-modulation of VEGF gene expression can be achieved *via* TNFSF15-DR3-mediated activation of the JNK-GATA3 signaling pathway that leads to miR-29b upregulation. These findings are of significance in aiding the development of new approaches to interfere with VEGF-driven neovascularization in disease conditions such as in cancers.

## MATERIALS AND METHODS

### Cell culture

Mouse endothelial cell line bEnd.3 were maintained in DMEM supplemented with 10% FBS and 1% penicillin/streptomycin (complete media) and incubated at 37°C and 5% CO_2_.

### Mice

Six-to 8-week-old female C57BL/6J mice were purchased from Academy of Military Medical Science (Beijing, China). Studies using experimental animals were performed according to protocols approved by Nankai University Ethics Committee on Pre-Clinical Studies.

### MicroRNA transfection

Scramble miRNA mimics (miR-Ctr, #24), miR-29b mimics (miR-29b, #miR10000127), scramble anti-miRNA mimics (anti-Ctr, #22) and anti-miR-29b mimics (anti-miR-29b, #miR20000127) were purchased from Ribobio (Guangzhou, China). Before transfection, bEnd.3 cells were seeded in 12-well plates (80000 cells per well) and cultured in Dulbecco's modified Eagle's medium (DMEM) containing 10% FBS for 12 hrs. For transient overexpression of miR-29b, cells was transfected with 50 nM miR-29b or miR-Ctr using Lipofectamine 2000 (#11668-019, Invitrogen, Carlsbad, CA, USA). For transient down-regulaton of miR-29b, cells were transfected with 100 nM anti-miR-29b or anti-Ctr, according to the manufacturer's instructions. Samples were collected after 24 hrs for quantification of miRNA or protein expression.

### Construction of bEnd.3-control cell line, bEnd.3-miR-29b cell line and bEnd.3-Zip-miR-29b cell line

The GFP-expressing lentivectors encoding miR-29b or Zip-miR-29b, miR-control and polybrene were purchased from GenePharma (Shanghai, China). The lentivector stably knockdown miR-29b (Zip-miR-29b) expression contained the following shRNA sequence: 5'- GATCCGTAGCACCATGAAATCAGTGTTTCAA GAGAACACTGATTTCAAATGGTGCTACTTTTTTG- 3'. Prior to transfection, bEnd.3 cell were seeded in six-well plates (2×10^5^ cells per well) and incubated overnight, then transduced with lentiviral supernatants containing lentiviral vectors coding for the miR-29b, Zip-miR-29b, or miR-control, respectively, and 5μg/mL polybrene at room temperature for 24 hrs. Culture media were then removed and replaced with fresh DMEM containing 10% FBS and incubated for 72 hrs in the presence of puromycin (10μg/mL) for selection. The transduction efficiency was monitored by qPCR for the miR-29b expression.

### Luciferase assays

For 3'-untranslated region (3'-UTR) assays, human embryonic kidney cell line 293T cells were co-transfected with miR-Ctrl or miR-29b mimics (50 nM final concentration), firefly luciferase reporter constructs containing WT or mutated 3'-UTR of VEGF using Lipofectamine 2000 reagent (Invitrogen, Carlsbad, CA, USA), and a renilla luciferase reporter vector to normalize the transfection efficiency. For the Dual-Luciferase^®^ Reporter Assay System, renilla and firefly luciferase activities were measured 24 hrs post transfection using the Dual-Luciferase Reporter System E1910 and a GloMax luminometer E6501 (Promega, Madison, USA). The luciferase activities represent the firefly/renilla luciferase ratios, and transfection efficiency was normalized to the control luciferase.

### Quantitative real-time polymerase chain reaction

MicroRNA isolation kit and Trizol were purchased from Qiagen (Valencia, CA, USA). MiR-29b RT Primer (#ssD1301049265), miR-29b Forward Primer (#ssD1301049267), miR-Reverse Primer (#ssD089261711), U6-RT Primer (#ssD0904071008), U6-Forward Primer (#ssD0904071006) and U6-Reverse Primer (#ssD0904071007) were purchased from Ribobio (Guangzhou, China). Cells were collected and homogenized in Trizol, total RNA including miRNAs extracted, and reverse transcription were performed according to the manufacturer's protocol. For PCR amplification of the cDNA fragment encoding targeted genes, the sense and antisense primer sequences for VEGF, GATA3, and β-actin were, respectively, 5′-GGCGATTTAG CAGCAGATAT AAGAA-3′ and 5′-GGAGATCCTT CGAGGAGCAC TT-3, 5′-CCATTACCAC CTATCCGCCC-3′ and 5′-CACACTCCCT GCCTTCTGTG, 5′-CAGAAGGAGA TTACTGCTCT-3′ and 5′-TACTCCTGCT TGCTGATCCA CATC-3.

### ELISA

Mouse VEGF ELISA Kit was purchased from R&D Systems (Minneapolis, MA, USA). The assays were carried out according the manufacturer's instructions. The absorbance was measured at 450nm with an iMark^TM^ Microplate Reader (Bio-Rad Laboratories, Hercules, CA, USA).

### Western blotting

Cells were homogenized and subjected to SDS/PAGE. The proteins were transferred to a polyvinylidene difluoride membrane (Roche Molecular Biochemicals, Quebec, Canada), blocked with 5% nonfat milk powder in TBS-T buffer (20 mM Tris-HCl, pH 7.4, 137 mM NaCl, and 0.1% Tween) for 1 hour at RT, incubated overnight at 4°C with primary antibody against the target proteins and then incubated with appropriate HRP-conjugated secondary antibodies. The films were developed with the ECL System (Millipore, Billerica, MA, USA). VEGF (#sc-152, 1:1000), GATA-3(#sc-9009, 1:1000) were purchased from Santa Cruz Biotechnology (Santa Cruz, CA, USA). DR3 (1:3000) was purchased from Sigma (St Louis, MO, USA). JNK(#9258S, 1:1000), phospho-JNK(#4668S, 1:1000), c-Jun(#9165S, 1:1000), phospho-c-Jun (#3270S, 1:1000) were obtained from Cell Signaling Technology (Danvers, MA).

### RNA interference

The siRNA for DR3 and GATA3 were purchased from Santa Cruz Biotechnology (Santa Cruz, CA, USA). Scrambled siRNA control was purchased from GenePharma (Shanghai, China). The sense and antisense sequences of the scrambled siRNA were as follows: 5′-UUCUCCGAAC GUGUCACGUT T-3′ and 5′-ACGUGACACG UUCGGAGAAT T-3. Cells were transfected with siRNAs using Lipofectamine 2000 reagent (Invitrogen, Carlsbad, CA, USA). Messenger RNA and protein levels of the target gene products were determined 24 hrs post transfection or as indicated in the text.

### *In vitro* angiogenesis assay

Forty-eight-well plates were coated with 40 μL of Matrigel (R&D Systems) and let to solidify for 30 min at 37°C. The bEnd.3 cells stably transfected miR-control or miR-29b mimics, anti-control or anti-miR-29b mimics were seeded (3×10^4^ cells/well) on top of the solidified Matrigel and incubated at 37°C for 24 hrs. The cells were stained with 3 μM calcein-AM (Invitrogen) for 30 min at 37°C and 5% CO_2_. Formation of the capillary tubule structures was observed and digitally photographed under an inverted light microscope at 5× magnification (Axiovert 200M; Zeiss, Oberkochen, Germany). Tubule lengths and areas were quantified by using Image-Pro Plus 6.0 software (Media Cybernetics, Rockville, MD, USA).

### *In vivo* angiogenesis assay

The bEnd.3 cells transduced with lentivirus encoding miR-ctr, miR-29b or Zip-miR-29b, respectively, were resuspended on ice in phenol red-free Matrigel solution and implanted into female C57BL/6J mice by s.c. injection (200 μL) in the abdominal region. Matrigel plugs were retrieved 14 days post implantation, photographed with a Leica M165FC stereoscopic microscope (Leica, Wetzlar, Germany). The plugs were each divided into three portions for hematoxylin and eosin (H&E) staining, fluorescent immunostaining, and Western blotting. For H&E staining, the plugs was fixed with 4% paraformaldehyde, paraffin embedded, and sectioned (5 μm). The sections were subjected to H&E staining. Red blood cell-containing luminal structures on the sections were counted under a microscope (Nikon 50i; Nikon, Tokyo, Japan). Three fields (magnification, 100×) per section were examined. For immunostaining, the plugs were fresh-frozen in OCT embedding medium, then sectioned (10 μm), The sections were equilibrated, hydrated with phosphate buffered saline (PBS) for 5 min at RT, and fixed in 4% paraformaldehyde for 15 min at RT, rinsed with PBS, blocked with 5% BSA for 1 hr at RT, and then incubated at 4°C overnight with anti-CD31 antibody (#553370, BD Biosciences, San Jose, CA, USA), then with Alexa Fluor 555-conjugated secondary antibody (#A-21434, Thermo Scientific, Rockford, IL, USA) for 1 hr at RT, mounted with DAPI Mounting Medium (Vector Laboratories, Burlingame, CA), and analyzed by using a Leica TCS SP5 confocal microscope (Leica, Wetzlar, Germany).

### Cell migration assays

Eight μm-pore Transwells were used (Corning Inc, Corning, NY, USA). Cells (5000/well) were seeded in the inserts pre-equilibrated with DMEM containing 10% FBS. The inserts were then placed in the culture wells and incubated for 24 hrs, rinsed, fixed in 4% paraformaldehyde for 10 min, and then subjected to crystal violet (Beyotime, Haimen, Jiangsu, China) staining. Digital images (10× magnification) of the underside of the inserts were taken with a microscope (ECLIPSE TS100, Nikon, Tokyo, Japan). The numbers of cells on six randomly selected fields per well per insert were counted.

### Statistical analysis

The data were subjected to variance analysis (ANOVA), followed by 2-tailed, unpaired Student's *t*-tests. Differences between two groups with p-values less than 0.05 were considered to be statistically significant.

## SUPPLEMENTARY MATERIAL


