# Supplementary material for: TNFSF15 suppresses VEGF production in endothelial cells by stimulating miR-29b expression via activation of JNK-GATA3 signals
Source: Oncotarget. 2016 Aug 29;7(43):69436–49. doi: 10.18632/oncotarget.11683 (PMC5342489; doi:10.18632/oncotarget.11683)
Supplement: Supplementary file 1 [file oncotarget-07-69436-s001.pdf]

# TNFSF15 suppresses VEGF production in endothelial cells by stimulating miR-29b expression *via* activation of JNK-GATA3 signals

**Supplementary Material**

MATERIALS AND METHODS

Transfection of siRNA into bEnd.3 cells. The siRNA (control or JNK specific) were purchased from GenePharma (Shanghai, China). The sense and antisense sequences of the scrambled siRNA and JNK-specific siRNA were as follows: 5′-UUCUCCGAAC GUGUCACGUT T-3′ and 5′-ACGUGACACG UUCGGAGAAT T-3, 5’-AAAGAAUGUC CUACCUUCUTT-3’ and 5’-AGAAGGUAGG ACAUU CUUUTT-3’[1]. Cells were transfected with siRNAs using Lipofectamine 2000 reagent (Invitrogen, Carlsbad, CA, USA). Messenger RNA and protein levels of the target gene products were determined 24 hrs post transfection.

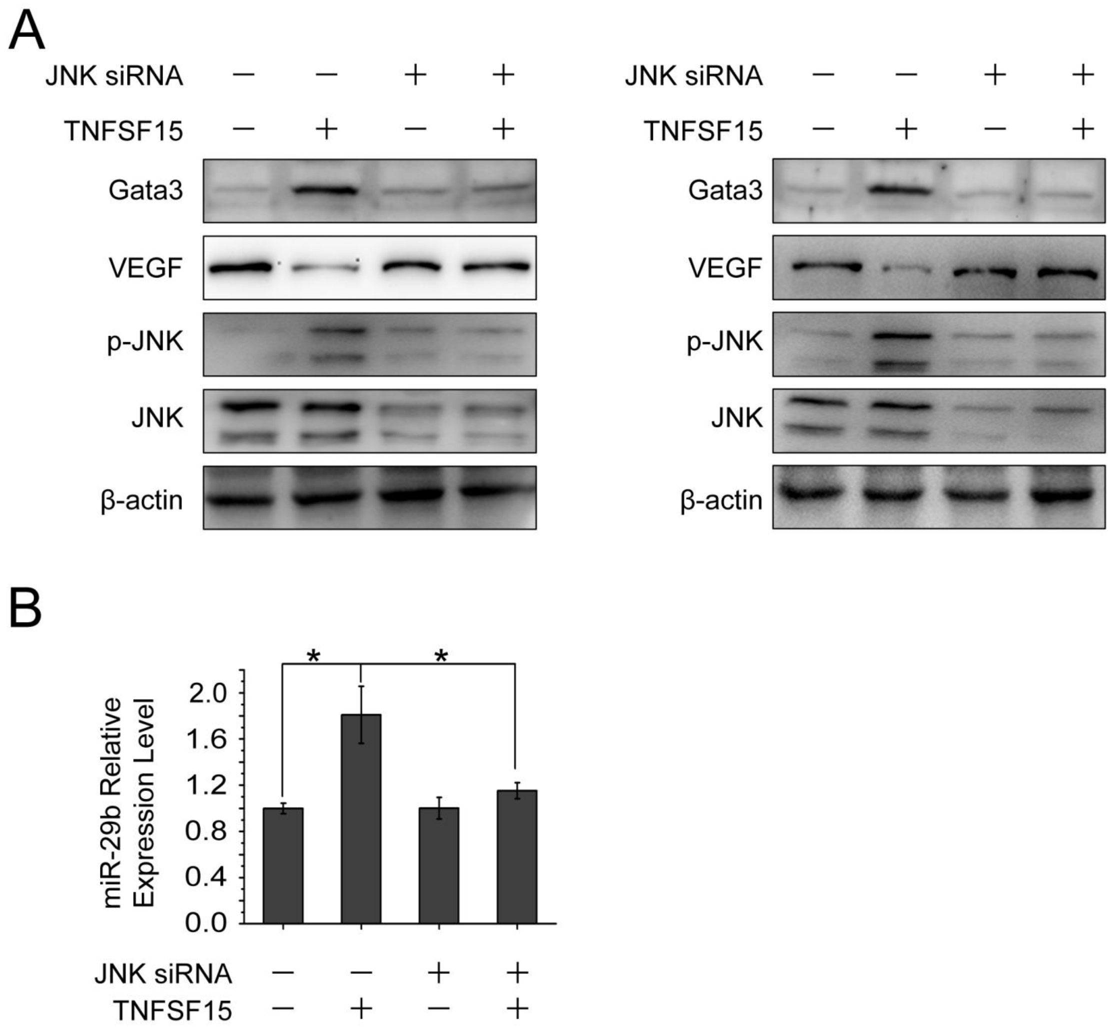


Figure S1: Effects of JNK gene-silencing on TNFSF15-stimulated GATA3 and miR-29b expression. (A) Duplicated experiments showing the effect of JNK siRNA (44 nM) on VEGF protein levels and GATA3 up-regulation in TNFSF15-treated bEnd.3 cells. (B) Changes of miR-29b levels following TNFSF15 treatment (24 hrs) in the presence or absence of JNK siRNA (44 nM). Data are mean±SD. *p <0.05; one-way ANOVA.

REFERENCES

1. Li G, Xiang Y, Sabapathy K and Silverman RH. An apoptotic signaling pathway in the interferon antiviral response mediated by RNase L and c-Jun NH2-terminal kinase. J Biol Chem. 2004; 279(2):1123-1131.
